# Supplementary material for: NDUFA4L2 rescues hyperoxia-induced migration defects in retinal endothelial cells by reversing isocitrate dehydrogenase flux blockade
Source: bioRxiv. 2026 Jul 15:2026.07.14.738274. Preprint. [Version 1] doi: 10.64898/2026.07.14.738274 (PMC13405267; doi:10.64898/2026.07.14.738274)

bioRxiv preprint doi: https://doi.org/10.64898/2026.07.14.738274; this version posted July 15, 2026. The copyright holder for this preprint (which was not certified by peer review) is the author/funder, who has granted bioRxiv a license to display the preprint in perpetuity. It is made available under a CC-BY 4.0 International license.

Supplemental Figure S1. NDUFA4L2 localizes in the artery of the P12 mouse retina

a) Representative P12 retinal flatmounts of C57BL6J (n=6) mice immunostained for isolectin b4 and NDUFA4L2, overlayed image showing NDUFA4L2 vascular expression pattern. Arrows indicate arteries (in red) and veins (in blue). b) Representative P12 retinal flatmounts of C57BL6J (n=6) mice immunostained for isolectin b4, NDUFA4L2, and alpha-smooth muscle actin (αSMA), showing NDUFA4L2 vascular expression pattern in arteries (red arrow) versus veins (blue arrow). Scale bar: A is 200 μm and B is 75 μm.

Supplemental Figure S2. MG132 and Roxadustat induced the transcription levels of HIF1α target genes, indicating active HIF1α in these conditions.

a) Quantitative RT-PCR (qRT-PCR) result showed that the low dose of MG132 treatment in primary HRECs increased the transcription levels of *NDUFA4L2* in normoxia and some HIF1α downstream targets. b) qRT-PCR of HIF1α downstream targets showed higher expression in response to Roxadustat treatment. Data are presented as mean ± SD (n = 3 per group). Statistical significance was determined using an unpaired two-tailed Welch's t-test.

bioRxiv preprint doi: https://doi.org/10.64898/2026.07.14.738274; this version posted July 15, 2026. The copyright holder for this preprint (which was not certified by peer review) is the author/funder, who has granted bioRxiv a license to display the preprint in perpetuity. It is made available under a CC-BY 4.0 International license.

Statistical significance is indicated as: *p-value ≤ 0.05, and **p-value ≤ 0.01. NS indicates no significant difference. ANGPTL4, Angiopoietin-like 4; PDK1, Pyruvate dehydrogenase kinase 1

Supplemental Figure S3. We didn't find NDUFA4L2 protein regulation by VEGF-VEGFR2 signaling in our analysis, but its transcription levels were changed by VEGF dependent manner.

a) Knockdown of VEGFR2 in primary HRECs showed very strong downregulation of VEGFR2 protein but did not change NDUFA4L2 protein levels, regardless of human VEGF-165 treatment. b) quantification of VEGFR2 levels from siVEGFR2 western blot showing strong downregulation of VEGFR2 protein. c) quantification of NDUFA4L2 levels from siVEGFR2 western blot showing no statistically significant differences. d) Supplementing media with different concentrations of VEGF-165 did not alter NDUFA4L2 protein levels. e) quantification of NDUFA4L2 levels from VEGF-165-treated primary HRECs showing no statistically significant differences. Data are presented as mean ± SD (n = 3 per group). Comparisons among four groups were analyzed using one-way ANOVA followed by Tukey's multiple comparisons post hoc test. Statistical significance is indicated as: ****p-value ≤ 0.0001. NS indicates no significant difference. f) Re-analyzed data from GEO dataset GSE221861 by Rameshekar et al.[48] showed the induction of *NDUFA4L2* transcription level in primary HRECs in response to VEGFA, but not its paralog *NDUFA4,* indicating that this may be regulated at transcriptional levels but not at protein levels. Data are presented as mean ± SD (n = 3 per group).

Supplemental Figure S4. NDUFA4L2 has some unique amino acids in its sequence compared to its paralog, NDUFA4, and these may be regulatory.

a) Protein sequences of NDUFA4L2 and NDUFA4 from Uniprot and aligned them in Clustal Omega, showing some regions of dissimilarity between the two

Supplemental Figure S5. NDUFA4L2 is a vertebrate-specific paralog restricted to organisms bearing vasculature.

a) The ortholog table generated using the PANTHER database shows species in which an NDUFA4L2 ortholog is present. Least diverged orthologs (LDO) and orthologs (O) were identified across mammals, a monotreme, a reptile, and multiple fish species, all of which possess a circulatory vascular system of any kind. b) Gene tree from the PANTHER database shows the evolutionary relationship between NDUFA4L2 (subfamily SF5) and its paralog NDUFA4 (subfamily SF4). NDUFA4L2 orthologs form a distinct vertebrate-specific clade (red), separate from the ancestral ND-MLRQ gene found in invertebrate species lacking a

bioRxiv preprint doi: https://doi.org/10.64898/2026.07.14.738274; this version posted July 15, 2026. The copyright holder for this preprint (which was not certified by peer review) is the author/funder, who has granted bioRxiv a license to display the preprint in perpetuity. It is made available under a CC-BY 4.0 International license.

closed vasculature (*Anopheles gambiae*, *Drosophila melanogaster*). Gene ID, gene name, and organism are shown for each node.

Supplemental Figure S6. IDH flux is not impacted in proliferating sub-confluent HRECs and shows no significant changes in response to NDUFA4L2 overexpression

a) GC-MS result indicates that NDUFA4L2 did not restore citrate and αKG in proliferating sub-confluent HRECs. Data are presented as mean ± SD (n = 3 per group). Comparisons among four groups were analyzed using one-way ANOVA followed by Tukey's multiple comparisons post hoc test. NS indicates no significant difference. Legends: NC, normoxia mCherry OE; NN, normoxia NDUFA4L2 OE; HC, hyperoxia mCherry OE; HN, hyperoxia NDUFA4L2 OE.

bioRxiv preprint doi: https://doi.org/10.64898/2026.07.14.738274; this version posted July 15, 2026. The copyright holder for this preprint (which was not certified by peer review) is the author/funder, who has granted bioRxiv a license to display the preprint in perpetuity. It is made available under a CC-BY 4.0 International license.

Supplemental Figure S1

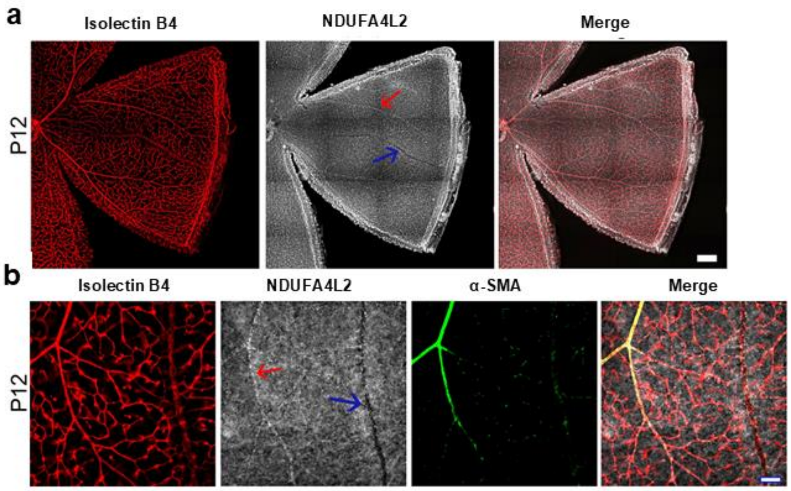

bioRxiv preprint doi: https://doi.org/10.64898/2026.07.14.738274; this version posted July 15, 2026. The copyright holder for this preprint (which was not certified by peer review) is the author/funder, who has granted bioRxiv a license to display the preprint in perpetuity. It is made available under a CC-BY 4.0 International license.

Supplemental Figure S2

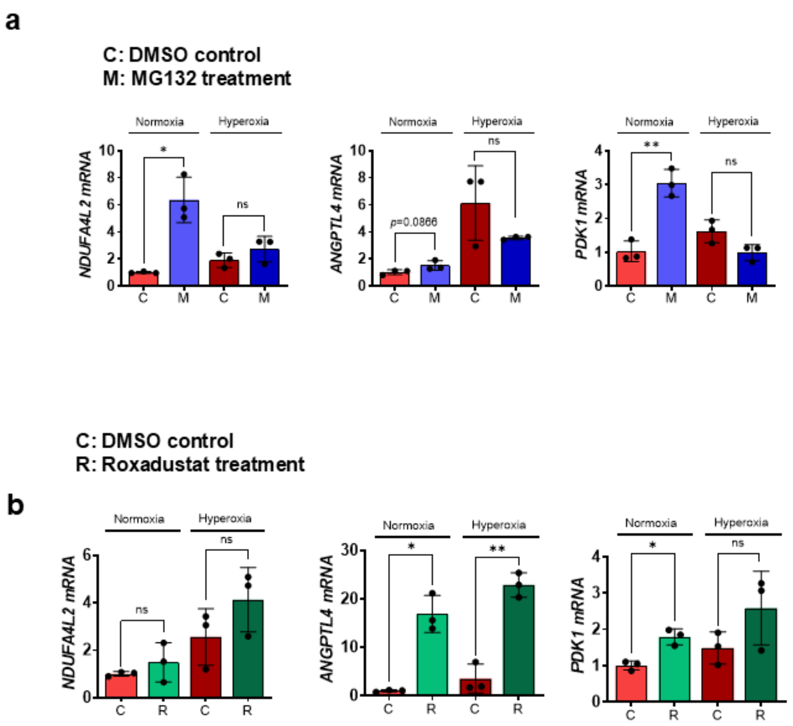

bioRxiv preprint doi: https://doi.org/10.64898/2026.07.14.738274; this version posted July 15, 2026. The copyright holder for this preprint (which was not certified by peer review) is the author/funder, who has granted bioRxiv a license to display the preprint in perpetuity. It is made available under a CC-BY 4.0 International license.

Supplemental Figure S3

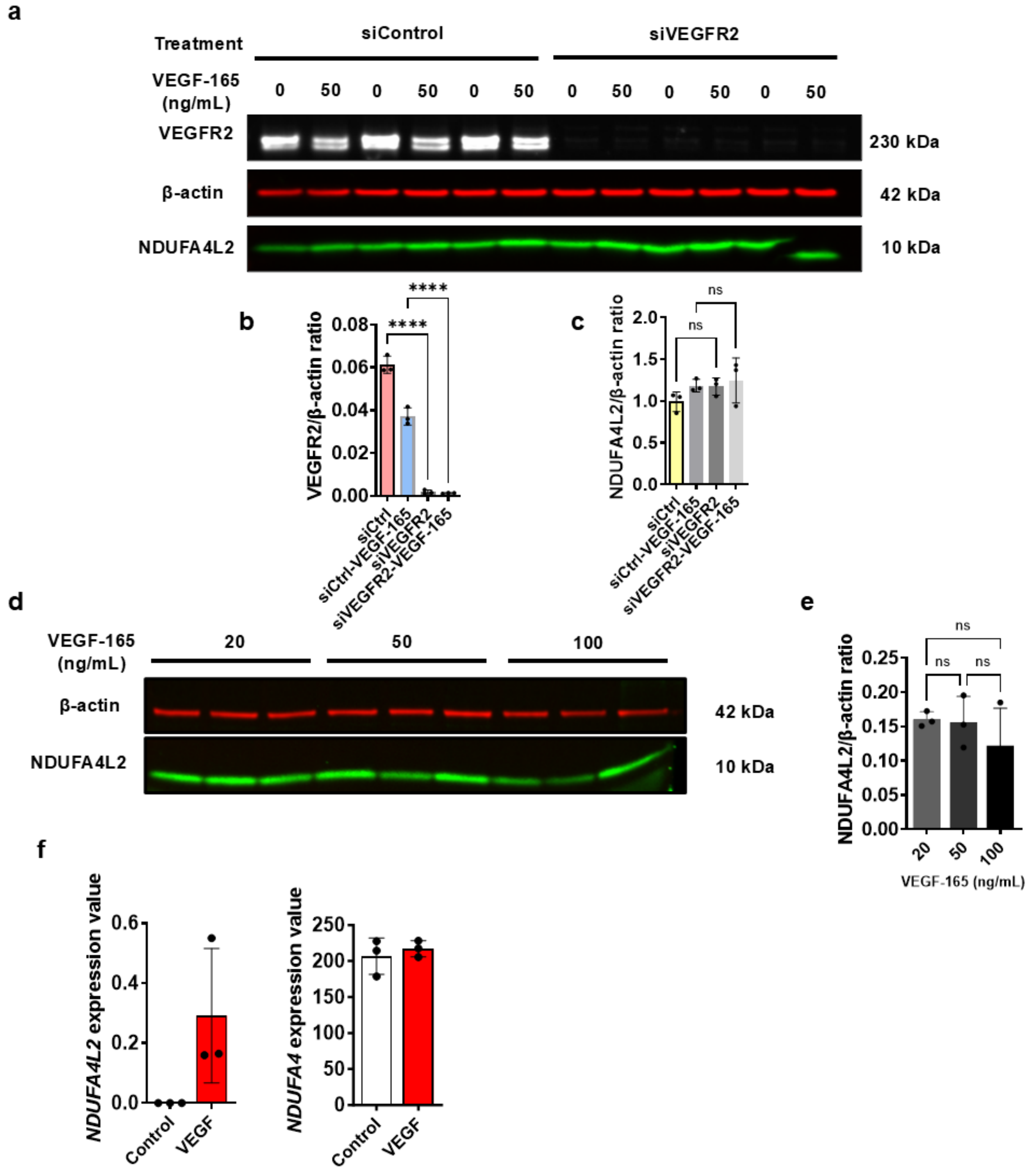

bioRxiv preprint doi: https://doi.org/10.64898/2026.07.14.738274; this version posted July 15, 2026. The copyright holder for this preprint (which was not certified by peer review) is the author/funder, who has granted bioRxiv a license to display the preprint in perpetuity. It is made available under a CC-BY 4.0 International license.

Supplemental Figure S4

a

Cluster Omega
NDFUA4 vs NDUFA4L2

```
4      -----MLRQIIGQAKKHPSLIPLFVFIGTGATGATLYLLRLALFNPDVCWDR-NNPEPWN   54
4L     MAGASLGARFYRQIKRHPGIIPMIGLICLGMGSAALYLLRLALRSPDVCWDRKNNPEPWN   60
            :  ::  * *:**.:**:: :*  *  .*:******** .******* *******

4      KLGPNDQYKFYSVNVDYSKLKKERPDF   81
4L     RLSPNDQYKFLAVSTDYKKLKKDRPDF   87
       :*.******* :*..**.****:****
```

bioRxiv preprint doi: https://doi.org/10.64898/2026.07.14.738274; this version posted July 15, 2026. The copyright holder for this preprint (which was not certified by peer review) is the author/funder, who has granted bioRxiv a license to display the preprint in perpetuity. It is made available under a CC-BY 4.0 International license.

Supplemental Figure S5

a

ORTHOLOGS

ID	Organism	Type
HUMAN|HGNC=29836|UniProtKB=Q9NRX3	Homo sapiens	LDO
PANTR|Ensembl=ENSPTRG00000045465.1|UniProtKB=A0A2J8KWA5	Pan troglodytes	LDO
GORGO|Ensembl=ENSGGOG00000010249.3|UniProtKB=G3R413	Gorilla gorilla gorilla	LDO
MACMU|Ensembl=ENSMMUG00000021786.4|UniProtKB=F7H7R8	Macaca mulatta	LDO
RAT|RGD=2324558|UniProtKB=D3ZXX8	Rattus norvegicus	LDO
BOVIN|Ensembl=ENSBTAG00000031503|UniProtKB=Q3SZ44	Bos taurus	LDO
PIG|Ensembl=ENSSSCG00000027621.4|UniProtKB=F1SL75	Sus scrofa	LDO
HORSE|Ensembl=ENSECAG00000023525.4|UniProtKB=A0A5F5PV33	Equus caballus	LDO
FELCA|Ensembl=ENSFCAG00000000189.4|UniProtKB=M3VU93	Felis catus	LDO
CANLF|Ensembl=ENSCAFG00845010422.1|UniProtKB=A0A8I3RXX3	Canis lupus familiaris	LDO
ORNAN|Ensembl=ENSOANG00000044477.1|UniProtKB=A0A6I8NQT7	Ornithorhynchus anatinus	LDO
ANOCA|Ensembl=ENSACAG00000040137.1|UniProtKB=A0A803TMJ4	Anolis carolinensis	LDO
ORYLA|Ensembl=ENSORLG00000005542.2|UniProtKB=H2LLR2	Oryzias latipes	O
ORYLA|Ensembl=ENSORLG00000003811.2|UniProtKB=H2LFK5	Oryzias latipes	LDO
DANRE|ZFIN=ZDB-GENE-130530-919|UniProtKB=A0A286YA77	Danio rerio	O
DANRE|ZFIN=ZDB-GENE-070912-358|UniProtKB=A0A0R4IT01	Danio rerio	LDO
LEPOC|Ensembl=ENSLOCG00000006035.1|UniProtKB=W5MFY1	lepisosteus oculatus	LDO

b

Tree Attribute Viewer

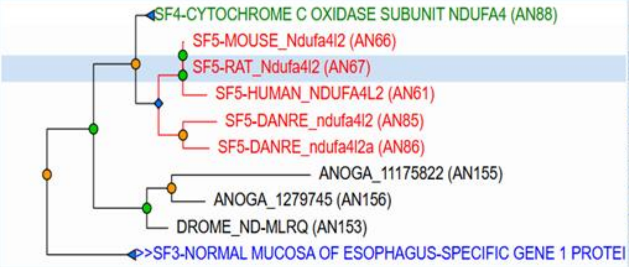

Tree	Gene Id	Gene Name	Organism
SF4-CYTOCHROME C OXIDASE SUBUNIT NDUFA4 (AN88)			
SF5-MOUSE_Ndufa4l2 (AN66)	MGI:3039567	Ndufa4l2	Mus musculus
SF5-RAT_Ndufa4l2 (AN67)	RGD:2324558	Ndufa4l2	Rattus norvegicus
SF5-HUMAN_NDUFA4L2 (AN61)	HGNC:29836	NDUFA4L2	Homo sapiens
SF5-DANRE_ndufa4l2 (AN85)	ZFIN:ZDB-GENE-130530-919	ndufa4l2	Danio rerio
SF5-DANRE_ndufa4l2a (AN86)	ZFIN:ZDB-GENE-070912-358	ndufa4l2a	Danio rerio
ANOGA_11175822 (AN155)	EnsemblGenome:AGAP013...	11175822	Anopheles gambiae
ANOGA_1279745 (AN156)	EnsemblGenome:AGAP003...	1279745	Anopheles gambiae
DROME_ND-MLRQ (AN153)	FlyBase:FBgn0052230	ND-MLRQ	Drosophila melanog...
>>SF3-NORMAL MUCOSA OF ESOPHAGUS-SPECIFIC GENE 1 PROTEI			

bioRxiv preprint doi: https://doi.org/10.64898/2026.07.14.738274; this version posted July 15, 2026. The copyright holder for this preprint (which was not certified by peer review) is the author/funder, who has granted bioRxiv a license to display the preprint in perpetuity. It is made available under a CC-BY 4.0 International license.

Supplemental Figure S6

GC-MS result

a

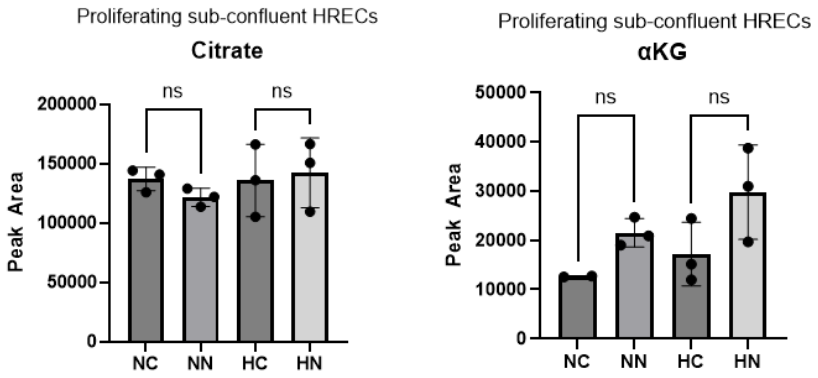

Supplement: 1 [file NIHPP2026.07.14.738274v1-supplement-1.pdf]
